# Low oxygen tension enhances the generation of lung progenitor cells from mouse embryonic and induced pluripotent stem cells

**DOI:** 10.14814/phy2.12075

**Published:** 2014-07-17

**Authors:** Elena Garreta, Esther Melo, Daniel Navajas, Ramon Farré

**Affiliations:** 1 Facultat de Medicina, Unitat de Biofísica i Bioenginyeria, Universitat de Barcelona, Barcelona, Spain; 2 CIBER de Enfermedades Respiratorias, Madrid, Spain; 3 Institut Investigacions Biomediques August Pi i Sunyer (IDIBAPS), Barcelona, Spain; 4 Institut de Bioenginyeria de Catalunya, Barcelona, Spain; 5 Centre de Medicina Regenerativa de Barcelona (CMRB), Parc de Recerca Biomèdica de Barcelona (PRBB), Dr. Aiguader88 7ª Planta, Barcelona, 08003, Spain; 6 F. Hoffmann‐La Roche, AG, NORD DTABldg. 69/331, Basel, CH‐4070, Switzerland

**Keywords:** Decellularization, embryonic stem cells, hypoxia, induced pluripotent stem cells, lung bioengineering, lung differentiation, lung progenitors, Nkx2.1, oxygen tension

## Abstract

Whole‐organ decellularization technology has emerged as a new alternative for the fabrication of bioartificial lungs. Embryonic stem cells (ESC) and induced pluripotent stem cells (iPSC) are potentially useful for recellularization since they can be directed to express phenotypic marker genes of lung epithelial cells. Normal pulmonary development takes place in a low oxygen environment ranging from 1 to 5%. By contrast, in vitro ESC and iPSC differentiation protocols are usually carried out at room‐air oxygen tension. Here, we sought to determine the role played by oxygen tension on the derivation of Nkx2.1+ lung/thyroid progenitor cells from mouse ESC and iPSC. A step‐wise differentiation protocol was used to generate Nkx2.1+ lung/thyroid progenitors under 20% and 5% oxygen tension. On day 12, gene expression analysis revealed that Nkx2.1 and Foxa2 (endodermal and early lung epithelial cell marker) were significantly upregulated at 5% oxygen tension in ESC and iPSC differentiated cultures compared to 20% oxygen conditions. In addition, quantification of Foxa2+Nkx2.1+Pax8‐ cells corresponding to the lung field, with exclusion of the potential thyroid fate identified by Pax8 expression, confirmed that the low physiologic oxygen tension exerted a significant positive effect on early pulmonary differentiation of ESC and iPSC. In conclusion, we found that 5% oxygen tension enhanced the derivation of lung progenitors from mouse ESC and iPSC compared to 20% room‐air oxygen tension.

## Abstract (precis)

Differentiation of pluripotent stem cells into lung progenitors is improved at low oxygen tension (5% oxygen) as compared with the conventional culture conditions of room air (20% oxygen).

## Introduction

Lung bioengineering is a regenerative medicine topic that has experienced a fast growth in the recent years (Soto‐Gutierrez et al. 2012). The proof of concept that the whole organ can be decellularized and subsequently recellularized has opened new perspectives in tissue engineering for the fabrication of potentially functional lungs (Song and Ott 2011; Badylak et al. 2012; Wagner et al. 2013), theoretically bypassing the problems of donor organ scarcity and transplant rejection. The major advantage of this approach is the fact that the 3D architecture and the biochemical composition of the resultant scaffold remain almost preserved, providing a microenvironment to the cells that is expected to be very similar to what they find in vivo in the native organ.

Several publications have already shown the potential of whole‐lung decellularization technology in terms of structure, composition, and mechanical properties of the organ scaffold (Cortiella et al. 2010; Ott et al. 2010; Petersen et al. 2010; Melo et al. 2013; Nonaka et al. 2013). Nevertheless, one important open question is to find appropriate cell sources to repopulate acellular lungs. Taking into account the diversity of cell phenotypes that are found in the adult lung (Morrisey and Hogan 2010; Garcia et al. 2012), it seems reasonable to coculture two or more different cell types in vitro, as it has already been tested by seeding a mixture of endothelial cells with neonatal or fetal lung cells into the lung scaffold (Ott et al. 2010; Petersen et al. 2010). However, it remains unclear whether these mature and fetal cells can be sufficiently expanded and can properly differentiate and function. Alternatively, pluripotent stem cells (either embryonic stem cells [ESC] or induced pluripotent stem cells [iPSC]) have been proposed for whole‐organ recellularization (Lau et al. 2012). Recent breakthroughs in the generation of iPSC from patients and their ability to differentiate into many specific cell types (Grskovic et al. 2011; Okano et al. 2013; Sommer and Mostoslavsky 2013) have established the feasibility of being a suitable cell source for acellular lung recellularization.

Early in embryonic development, the onset of expression of the homeodomain‐containing transcription factor Nkx2.1 in the ventral wall of the anterior foregut defines a primordial progenitor stage from which lung will develop (Morrisey and Hogan 2010). Thus, the transcription factor Nkx2.1 is the earliest marker of the specified lung endoderm (Morrisey and Hogan 2010). However, Nkx2.1 expression is not restricted to the lung, and is also found in the thyroid and the forebrain. Nkx2.1 knockout mice fail to properly develop thyroid and lung organs (Kimura et al. 1996; Yuan et al. 2000), as well as exhibit severe abnormalities in forebrain development (Kimura et al. 1996). Therefore, Nkx2.1 is considered a key transcriptional regulator in lung, thyroid, and forebrain development. Besides Nkx2.1 transcription factor, Gata and Foxa transcription factor families are also known to be required for early lung development (Morrisey and Hogan 2010). In mouse, Foxa2 is first expressed in the primitive streak at E6.5, and later in embryonic development, it is detected in organs derived from the foregut endoderm, such as the lung (Wan et al. 2005). Lung formation occurs in a region marked by coexpression of Nkx2.1 and Foxa2 (Bohinski et al. 1994), whereas thyroid organogenesis is marked by coexpression of Nkx2.1 and Pax8 (Di Palma et al. 2003).

Most ESC and iPSC differentiation strategies try to mimic in vitro the key signaling pathways that direct early lineage commitment in the embryo. In this way, it has been described that high levels of nodal signaling (induced by activin A) promote the generation of definitive endoderm (DE) from ESC and iPSC (Murry and Keller 2008). Posterior endodermal lineages, such as hepatic (Basma et al. 2009) and intestinal cells (Spence et al. 2011), have been shown to be easily derived from these DE cells. Interestingly, the most anterior endodermal lineages, such as lung epithelium and thyroid, have been challenging to derive (Ali et al. 2002; Rippon et al. 2004; Samadikuchaksaraei et al. 2006; Wang et al. 2007). Nevertheless, in recent works, researchers have reported significant advances in the efficient generation of anterior endoderm from ESC and iPSC and subsequent formation of differentiated lung cells (Green et al. 2011; Longmire et al. 2012; Mou et al. 2012; Ghaedi et al. 2013; Huang et al. 2014). Concretely, Longmire et al. (2012) used a Nkx2.1‐GFP mouse ESC reporter line to follow the course of specification of lung and thyroid progenitors. In their publication, Longmire et al. (2012) were able to define the signaling conditions required in vitro to preferentially differentiate lung and thyroid progenitors with very scarce contamination of Nkx2.1+ neuroectoderm. Following a stage‐specific inhibition of BMP and TGFβ signaling, they generated pure endodermal Nkx2.1+ populations competent to further differentiate into lung and thyroid cells (Longmire et al. 2012). In addition, they found that Nkx2.1+ cells were also expressing proteins known to be expressed in endodermal and early lung epithelial cells, such as Foxa2, Sox2, or Sox9 (Longmire et al. 2012).

In parallel, there is an increasing interest on mimicking the oxygen tension of in vivo cell microenvironments, in order to modulate cell proliferation, differentiation, and function (Simon and Keith 2008; Millman et al. 2009; Wion et al. 2009; Mohyeldin et al. 2010). Oxygen gradients are present in developing embryos, which experience much lower oxygen tensions (Simon and Keith 2008; Dunwoodie 2009; Gao and Raj 2010) than the one commonly used to culture cells in vitro (20% oxygen). Indeed, low oxygen tension has been shown to play a role in the differentiation of stem cells into many different fates such as cardiac cells (Ng et al. 2010; Van Oorschot et al. 2011; Horton and Auguste 2012), endothelial cells (Han et al. 2010; Prado‐Lopez et al. 2010; Shin et al. 2011), neuronal cells (Fernandes et al. 2010; Garita‐Hernández et al. 2013; Stacpoole et al. 2013; Binh et al. 2014), chondrogenic cells (Koay and Athanasiou 2008; Adesida et al. 2012), and hematopoietic cells (Lesinski et al. 2012). Recently, differentiation of human ESC into the oligodendrocyte lineage has been accomplished under physiologically relevant 3% oxygen conditions (Stacpoole et al. 2013). In addition, the generation of retinal progenitor cells from human ES and iPS cells (Bae et al. 2012) as well as the generation of photoreceptors from mouse ESC (Garita‐Hernández et al. 2013) are enhanced under 2% oxygen conditions. Thus, we hypothesized that the role of the oxygen partial pressure in the culture medium could be relevant in the differentiation of ESC and iPSC into lung epithelial cells.

Accordingly, here we describe the effect of using physiologic levels of oxygen tension on the generation of Nkx2.1+ lung/thyroid progenitors from mouse ESC and iPSC. We carried out a recently described step‐wise differentiation protocol (Longmire et al. 2012) that allows the derivation of Nkx2.1+ lung/thyroid progenitor cells with minimal contamination of Nkx2.1+ ectodermal cells. On day 12, we analyzed the expression of Nkx2.1 (lung/thyroid progenitor marker), Foxa2 (endodermal and early lung epithelial cell marker), Pax8 (thyroid cell marker), and Oct4 (pluripotent cell marker) by qPCR and immunofluorescence. Gene expression analysis revealed a significant increase in Nkx2.1 and Foxa2 gene expression in 5% compared to 20% oxygen conditions. Coexpression of Nkx2.1 and Foxa2 at the protein level was confirmed by immunofluorescence. Subsequent quantitative analysis by triple immunofluorescence against Foxa2, Nkx2.1, and Pax8 showed that 5% oxygen tension enhanced the generation of Foxa2+Nkx2.1+Pax8‐ lung progenitor cells form ESC and iPSC.

## Methods

### Pluripotent stem cell culture

Mouse embryonic stem cell (mESC) line E14Tg2A was kindly provided by A.E. Bishop from the faculty of Medicine of the Imperial College London. miPS cell line iPS‐wt4F (generated from mouse embryonic fibroblasts using the four original Yamanaka pMXs retroviral vectors) was kindly provided by J.C. Belmonte from the Center of Regenerative Medicine of Barcelona (CMRB) (Menendez et al. 2012). mESC and miPS cell lines were routinely maintained as colonies on a feeder layer of mitomycin‐inactivated mouse embryonic fibroblasts (iMEF) in basic murine embryonic stem cell medium (ESCM) composed of high‐glucose Dulbecco's modified Eagle's medium (DMEM, Gibco, Waltham, MA) supplemented with 15% (v/v) fetal bovine serum (Hyclone, Waltham, MA), 1% nonessential amino acids (Gibco), 1% sodium pyruvate (Gibco), 2 mmol/L L‐glutamine (Gibco), 0.1 mmol/L 2‐mercaptoethanol (Sigma‐Aldrich, St. Louis, MO), 100 U/mL penicillin/streptomycin (Gibco), and 1000 U/mL leukemia inhibitory factor (LIF, Chemicon, Billerica, MA). For differentiation experiments iMEF were removed from the cultures by passaging mESC and miPSC two times (1:5 dilution) to gelatin‐coated cell culture plates in mESCM with 2000 U/mL of LIF. mES and iPS cells were maintained under undifferentiated conditions at 20% oxygen tension and 5% CO_2_.

### Oxygen tension control

Oxygen conditions were created in a cell culture incubator with automatic control of oxygen and 5% CO_2_ concentrations (model 3141; Forma Scientific, Waltham, MA).

### Embryoid bodies formation

Embryoid bodies (EBs) were formed by using the hanging drop method. Briefly, a suspension of mESC or miPSC (3 × 10^4^ cells/mL) was plated in drops (25 *μ*L/drop) onto the lid of a Petri dish in mESCM without LIF. Stem cells were left to self‐aggregate for 3 days (72 h). To test the hypoxic effects on the initial stem cell differentiation, EBs from mouse ESC and iPSC were generated under 20% oxygen and 5% oxygen conditions. The number of formed EBs was quantified after 3 and 5 days by counting the number of aggregates formed from the same initially plated drops (25 *μ*L/drop, 3 × 10^4^ cells/mL). Additionally, the EBs adhesion was quantified by counting the number of attached EBs after 2 days of adherent culture onto gelatin‐coated plates (% of attached EBs = (number of adhered EBs/number of initially seeded EBs) × 100). For the adhesion experiments, we tested two different differentiation media consisting of DMEM (Gibco) supplemented with N2 (Gibco), B27 (Gibco), 0.5%, 2 mmol/L L‐glutamine (Gibco), 100 U/mL penicillin/streptomycin (Gibco), and 50 ng/mL Activin A (338‐AC‐050; R&D, Minneapolis, MN), either with addition of 0.5% (v/v) fetal bovine serum (Hyclone) (low‐serum medium) or without addition of fetal bovine serum (serum‐free medium).

### Generation of endodermal lung progenitors from pluripotent stem cells

**Figure 1.**
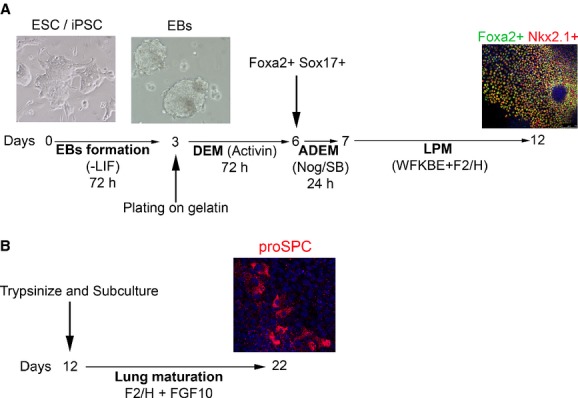
Timeline of the differentiation protocol. (A) A step‐wise differentiation protocol was used to generate Foxa2+Nkx2.1+ endodermal lung progenitors at 20% and 5% oxygen cell culture conditions. DEM = definitive endoderm medium; ADEM = anterior definitive endoderm medium; LPM = lung progenitors medium; Nog = Noggin; SB = SB431542; WFKBE = Wnt3a, FGF10, FGF7, BMP4, EGF; F2/H = FGF2, heparin sodium salt. (B) On day 12, cell cultures were gently trypsinized and subcultured. On day 22, cells were analyzed for the expression of mature lung markers, such as pro‐surfactant protein C (proSPC).

The differentiation protocol toward pulmonary progenitors was adapted from a previous published work (Longmire et al. 2012). Briefly, a step‐wise differentiation protocol was used to generate Foxa2+Nkx2.1+endodermal lung/thyroid progenitors at 20% and 5% oxygen cell culture conditions (Fig. 1A).

#### Stage 1: Induction of definitive endoderm

Embryoid bodies were formed from mES and iPS cells for 3 days (72 h) as explained above. Next, EBs were harvested and plated onto gelatine‐coated 12‐well plates (10‐15 EBs/well) in definitive endoderm induction medium (DEM) for 3 days (72 h). DEM was composed of DMEM (Gibco) supplemented with N2 (Gibco), B27 (Gibco), 0.5% (v/v) fetal bovine serum (Hyclone), 2 mmol/L L‐glutamine (Gibco), 100 U/mL penicillin/streptomycin (Gibco), and 50 ng/mL Activin A (338‐AC‐050; R&D).

#### Stage 2: Anteriorization of endoderm

On day 6, medium was changed to anterior definitive endoderm induction medium (ADEM) for 24 h. ADE medium was composed of DMEM (Gibco) supplemented with N2 (Gibco), B27 (Gibco), 0.5% (v/v) fetal bovine serum (Hyclone), 2 mmol/L L‐glutamine (Gibco), 100 U/mL penicillin/streptomycin (Gibco), 100 ng/mL mNoggin (1967‐NG‐025; R&D), and 10 μmol/L SB431542 (Sigma‐Aldrich).

#### Stage 3: Generation of lung progenitors

On day 7, medium was switched to lung progenitors induction medium (LPM) composed of DMEM (Gibco) supplemented with N2 (Gibco), B27 (Gibco), 0.5% (v/v) fetal bovine serum (Hyclone), 2 mmol/L L‐glutamine (Gibco), 100 U/mL penicillin/streptomycin (Gibco), 100 ng/mL mWnt3a, 10 ng/mL hFGF10, 10 ng/mL mFGF7, 10 ng/mL mBMP4, 20 ng/mL hEGF, 500 ng/mL mFGF2, and 100 ng/mL heparin sodium salt (Sigma, H4784). Growth factors were supplied by Peprotech. Cultures were differentiated for 5 days with periodic media changes.

In order to determine the effect of oxygen tension, differentiation of mES and iPS cells following this three‐stage differentiation protocol was performed under both 20% oxygen and 5% oxygen.

### Maturation of pluripotent stem cells‐derived lung progenitors

On day 12 of the above protocol, cell cultures were gently trypsinized and cells were replated onto gelatin‐coated 12‐well plates (50,000 cells/cm^2^). Cultures were grown for 10 days in DMEM (Gibco) supplemented with N2 (Gibco), B27 (Gibco), 0.5% (v/v) fetal bovine serum (Hyclone), 2 mmol/L L‐glutamine (Gibco), 100 U/mL penicillin/streptomycin (Gibco), 500 ng/mL mFGF2, 100 ng/mL hFGF10, and 100 ng/mL heparin sodium salt (Fig. 1B). Growth factors were supplied by Peprotech (Rocky Hill, NJ).

### Immunocytochemistry

For immunostainings, samples were fixed with 4% p‐formaldehyde (PFA) for 15 min, washed with PBS, and blocked with blocking solution for 1 h (1% BSA, 6% fetal bovine serum, 0.5% triton in TBS) and incubated overnight with primary antibodies. Mouse anti‐Oct4 (sc‐5279; Santa Cruz Biotechnologies, Dallas, TX), mouse anti‐Sox2 (mab2018; R&D), goat anti‐Foxa2 (R&R, AF2400), mouse anti‐Sox17 (MAB1924; R&D), rabbit anti‐Nkx2.1 (sc‐13040; Santa Cruz Biotechnologies, Dallas, TX), mouse anti‐Pax8 (Abcam, Cambridge, UK, ab53490), mouse anti‐Tuj1 (Sigma Aldrich, T8578), mouse anti‐(pan)cytokeratin (Sigma Aldrich, C2562), rabbit anti‐proSPC (Abcam, ab40879), and goat anti‐CC10 (sc‐9772; Santa Cruz Biotechnologies) were used as primary antibodies. After overnight primary antibody incubation, samples were washed with washing solution (0.1% BSA, 6% fetal bovine serum, 0.1% triton in TBS) and incubated with anti‐mouse (donkey)‐Alexa488, anti‐mouse (donkey)‐Dylight549, anti‐goat (donkey)‐Alexa488, anti‐mouse (donkey)‐Alexa647, anti‐rabbit (donkey)‐Cy3 secondary antibodies (all from Jackson Immunoresearch, Suffolk, UK). Samples were also counterstained with DAPI (#21490; Invitrogen, Carlsbad, CA). For quantification of Nkx2.1+Foxa2+Pax8‐immunostained cells on day 12, three differentiated samples per experiment were imaged using a Leica SP5 microscope. An average of 800 cells per image was counted using ImageJ (Bethesda, MA), and data points represent the average of three independent experiments.

### Isolation of RNA, Reverse Transcription (RT)‐PCR, and real‐time PCR analysis

RNA was extracted from cells using Trizol reagent (#15596‐026; Invitrogen), following the manufacturer protocol. All samples were treated with TURBO DNase (#AM2238; Ambion, Waltham, MA) to remove any residual genomic DNA and 1 *μ*g of RNA was used to synthesize cDNA using reverse transcription reagents (#4368813; Applied Biosystems, Carlsbad, CA). Approximately 25 ng of cDNA was used to quantify gene expression by using Premix Ex Taq (#RR390L; Takara, Saint‐Germain‐en‐Laye, France) in an ABI Prism 7300 thermocycler (Applied Biosystems). Relative gene expression, normalized to Gapdh control, was calculated using the 2^−ΔΔCT^ method to quantify fold change in gene expression of the indicated gene compared to baseline expression (fold change = 1) in ES or iPS cells. IDT Prime Time assays for Foxa2, Sox17, Nkx2.1, Pax8, and Oct4 genes were used as probes.

### Statistical analysis

Statistical comparisons between groups were performed by Student's *t*‐test, and *P *<**0.05 was considered as a statistically significant difference between groups.

## Results

### Efficiency of EBs formation

Mouse pluripotent stem cell lines, E14tg2a ESC line and iPSWT4F iPSC line, were maintained and passaged under undifferentiation state at 20% oxygen tension. Both pluripotent stem cell lines exhibited expression of pluripotency marker genes, such as Oct4 and Sox2, as expected (data not shown).

**Figure 2.**
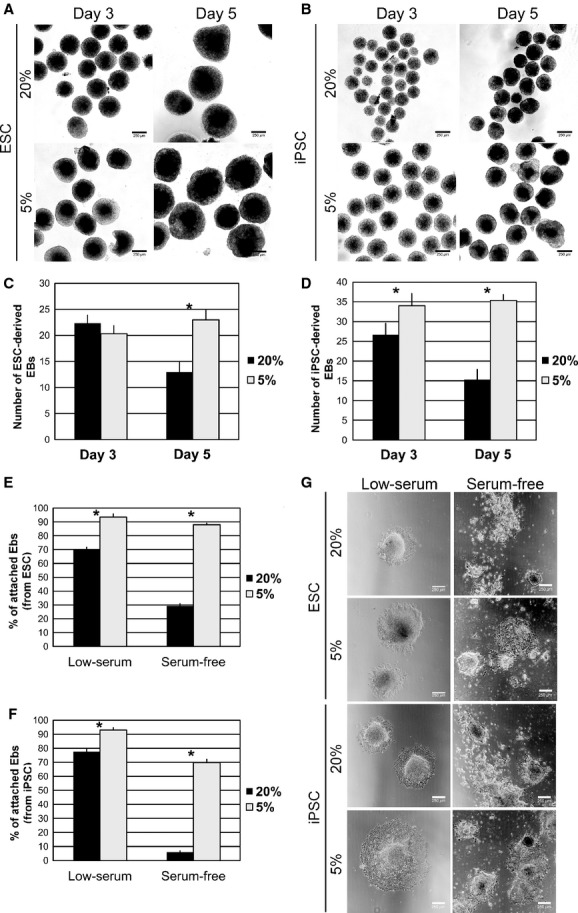
Low oxygen tension enhances the formation of EBs (embryoid bodies). Representative phase‐contrast images of EBs on day 3 and day 5 generated from (A) ESC and (B) iPSC, under 20% and 5% oxygen. Quantification of EBs generated from (C) ESC and (D) iPSC in suspension culture on day 3 and day 5 under 20% and 5% oxygen tensions. Percentage of EBs on day 3 generated from (E) ESC and (F) iPSC that adhered onto gelatin‐coated plates after 2 days in culture in the presence of 0.5% serum (low serum) or serum‐free supplemented medium and activin A under 20% and 5% oxygen tensions. (G) Representative phase‐contrast images of ESC‐ and iPSC‐derived cultures on day 5, after EB formation and adhesion onto gelatin‐coated plates for 2 days. Data collected from *N *=**3 independent experiments. Statistical analysis was performed by Student's *t*‐test and *P *<**0.05 (*) indicated significant difference between groups. ESC, embryonic stem cells; iPSC, induced pluripotent stem cells.

The formation of EBs is commonly performed as a first step in the majority of the differentiation protocols of pluripotent stem cells. Such cell aggregates are known to recapitulate in vitro early events of embryonic cell development. Also, EBs formation facilitates complex cell–cell interactions and the activation of endogenous signaling necessary for differentiation. Therefore, we sought to evaluate the effect of low oxygen levels on the efficiency of EB formation from mouse ESC and iPSC (ESC medium without LIF). Both ESC and iPSC formed dense simple EBs in both oxygen conditions (Fig. 2A and B). In ESC, the number of EBs formed after 3 days in suspension culture were similar at both 20% and 5% oxygen conditions (22.3 ± 1.5 vs. 20.3 ± 1.5, *n *=**3) (Fig. 2C). However, after 5 days in suspension culture the number of ESC‐derived EBs was significantly lower at 20% compared to 5% oxygen tension (13.0 ± 2.0 vs. 23.0 ± 2.0, *n *=**3, *P *<**0.05) (Fig. 2C). In iPSC, EB numbers after 3 days in suspension culture were significantly lower at 20% than 5% oxygen tension (26.6 ± 2.8 vs. 34.0 ± 3.0, *n *=**3, *P *<**0.05) (Fig. 2D), and after 5 days in suspension culture this difference was enlarged (15.3 ± 2.5 vs. 35.3 ± 1.5, *n *=**3, *P *<**0.05) (Fig. 2D). Further differentiation was induced by EBs adhesion into gelatin‐coated plates. The ability of EBs to adhere onto gelatin‐coated plates and to develop outgrowths is required in this protocol for their further differentiation. In addition, it is known that low serum or serum‐free supplementation and the presence of activin A are essential for the efficient generation of DE cells. Thus, the EBs adhesion capacity was evaluated in both oxygen conditions in the presence of 0.5% serum or in serum‐free medium. We found that ESC‐derived EBs in low‐serum medium attached in higher percentage at 5% compared to 20% oxygen conditions (93.5 ± 2.2% vs. 70.4 ± 1.2%, *n *=**3, *P *<**0.05) (Fig. 2E). The same tendency was observed in serum‐free medium, where the percentage of attached ESC‐derived EBs was significantly higher at 5% compared to 20% oxygen conditions (88.0 ± 0.9% vs. 29.3 ± 1.7%, *n *=**3, *P *<**0.05). In the case of iPSC‐derived EBs, also 5% oxygen tension enhanced EBs adhesion compared to 20% oxygen tension (92.9 ± 1.7% vs. 77.5 ± 2.1%, *n *=**3, *P *<**0.05) in low‐serum medium (Fig. 2F). In serum‐free medium, iPSC‐derived EBs adhesion was remarkably low at 20% oxygen tension (5.9 ± 2.3%), and there was also a statistically significant difference between 5% and 20% oxygen conditions (69.7 ± 2.3% vs. 5.9 ± 2.3%, *n *=**3, *P *<**0.05) (Fig. 2F). Attached EBs derived from ESC as well as iPSC were able to develop outgrowths in both oxygen and medium conditions (Fig. 2G). However, due to the poorer EBs attachment observed in serum‐free medium at 20% oxygen tension in ESC (29.3 ± 1.7%) (Fig. 2E) and iPSC (5.9 ± 2.3%) (Fig. 2F), we decided to carry out the subsequent pulmonary differentiation experiments in low‐serum medium.

Overall, these data suggested that physiological oxygen tension (5%) enhanced the generation of EBs from mouse ESC and iPSC.

### Effect of oxygen tension on the induction of definitive endoderm

After EBs formation, the next step on the differentiation toward the lung field is the induction of definitive endoderm cells (DE medium). Definitive endoderm was induced by exposing EBs to high concentrations of activin A in low‐serum supplemented medium for 3 days in adherent cultures (Fig. 1A).

**Figure 3.**
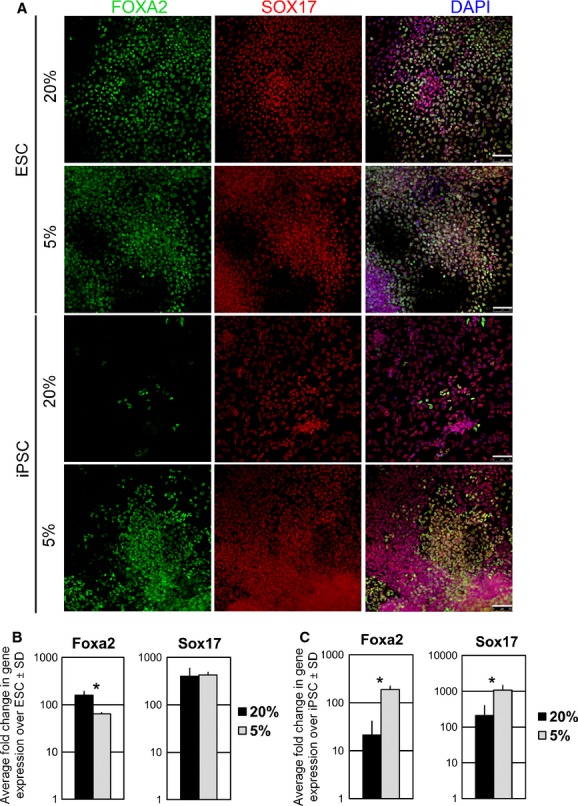
Induction of definitive endoderm (DE) under 20% and 5% oxygen tensions. (A) Representative immunofluorescence images of FOXA2 and SOX17 expression on day 6 of the protocol of ESC‐ and iPSC‐derived cells after DE induction under 20% and 5% oxygen tensions. Scale bar is 50 *μ*m. qPCR analysis of Foxa2 and Sox17 gene expression on day 6 of the protocol in (B) ES‐derived cells and (C) iPS‐derived cells, at 20% and 5% oxygen tensions. Data were collected from *N *=**3 independent experiments. Statistical analysis was performed by Student's *t*‐test and *P *<**0.05 (*) indicated significant difference between groups. ESC, embryonic stem cells; iPSC, induced pluripotent stem cells.

On day 6 of differentiation, ES and iPS‐derived cells stained positive for Foxa2 and Sox17, transcription factors distinctive of DE (Fig. 3A, ESC and iPSC). In ES‐derived cells, Foxa2+Sox17+cells were abundantly found in both oxygen conditions (Fig. 3A, ESC). In iPS‐derived cells, the amount of Foxa2+Sox17+ cells appeared to be surprisingly lower at 20% oxygen compared to 5% oxygen, in all the samples analyzed (Fig. 3A, iPSC).

In ESC differentiated cultures, Foxa2 gene expression was higher at 20% oxygen compared to 5% oxygen, while no significant change between 20% and 5% oxygen conditions was found for Sox17 gene expression (Fig. 3B). In the case of iPSC‐differentiated cultures, gene expression analysis revealed a significantly greater upregulation of Foxa2 and Sox17 at 5% compared to 20% oxygen tension (Fig. 3C).

Therefore, a DE cell population was generated in both oxygen conditions. However, in iPSC, differentiated cultures at 5% oxygen tension appeared to be more prone to generate DE cells.

### Effect of oxygen tension on the generation of Foxa2+Nkx2.1+ progenitors

Following DE induction, anteriorization of the foregut endoderm was accomplished by inhibition of the TGFβ and BMP signaling (ADE medium) (Longmire et al. 2012). Subsequent dorsoventral patterning of the anterior foregut endoderm yielded a population of Foxa2+Nkx2.1+ cells that potentially corresponded to the lung lineage (LP medium) (Longmire et al. 2012).

**Figure 4.**
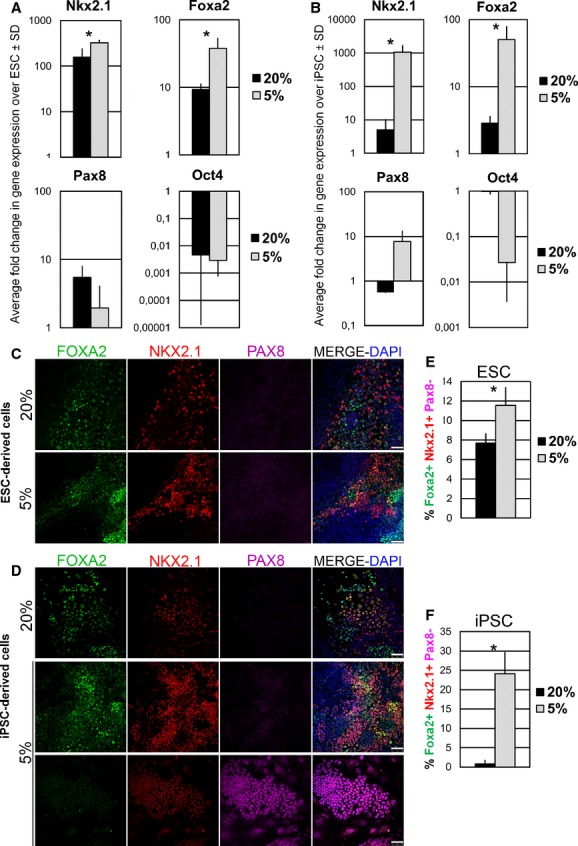
Low oxygen tension improves the generation of Foxa2+Nkx2.1+Pax8‐ lung progenitors. qPCR of Nkx2.1, Foxa2, Pax8, and Oct4 gene expression on day 12 in (A) ESC‐ and (B) iPSC‐differentiated cultures, under 20% and 5% oxygen tensions. Representative images of triple immunofluorescence on day 12 of the protocol against FOXA2, NKX2.1 and PAX8 in (C) ESC‐ and (D) iPSC‐differentiated cultures, under 20% and 5% oxygen conditions. Scale bar is 50 *μ*m. Quantification of FOXA2+NKX2.1+PAX8‐ cells derived from (E) ESC and (F) iPSC, on day 12, under 20% and 5% oxygen conditions. Data were collected from *N *=**3 independent experiments. Statistical analysis was performed by Student's *t*‐test and *P *<**0.05 (*) indicated significant difference between groups. ESC, embryonic stem cells; iPSC, induced pluripotent stem cells.

On day 12 of the differentiation process, gene expression analysis of ESC differentiated cultures revealed that Nkx2.1 and Foxa2 were significantly upregulated at 5% compared to 20% oxygen tension (Fig. 4A). Analysis of Pax8 expression was also performed as an indicator of thyroid lineage. Some Pax8 gene expression was detected in ESC differentiated cultures (less than 10‐fold upregulation over undifferentiated ESC) regardless of the oxygen conditions (Fig. 4A). In addition, a marked downregulation of Oct4 gene expression demonstrated that both oxygen conditions permitted the differentiation of ESC (Fig. 4A).

Day‐12 iPSC‐differentiated cultures followed a similar tendency. Nkx2.1 and Foxa2 were significantly upregulated at 5% compared to 20% oxygen tension (Fig. 4B). Pax8 gene expression was only scarcely detected in 5% oxygen differentiated cultures, while it was undetectable in 20% oxygen differentiated cultures (Fig. 4B). Interestingly, Oct4 gene expression was hardly downregulated in 20% oxygen compared to a noticeable downregulation in cultures at 5% oxygen (Fig. 4B). This observation suggested a presence of a pool of cells still expressing the pluripotency marker gene Oct4 and therefore indicating that iPSC at 20% oxygen tension could not properly differentiate.

Next, to prove the presence of a cell population coexpressing the markers of early lung epithelial cells, Nkx2.1 and Foxa2, but at the same time excluding the possible existence of thyroid progenitors characterized by Pax8 expression, we performed triple immunostainings for Nkx2.1, Foxa2, and Pax8. We found that ESC‐derived cells on day 12 contained a pool of cells coexpressing Nkx2.1 and Foxa2 transcription factors, both at 20% and 5% oxygen conditions. The great majority of Foxa2+Nkx2.1+ cells were negative for Pax8 (Fig. 4C, 20% and 5%). In the case of iPS‐derived cells, Foxa2+Nkx2.1+ cell clusters were easily found in samples differentiated at 5% oxygen tension, but were scarcely generated at 20% oxygen tension (Fig. 4D). Again, Pax8+ cells were very rare (Fig. 4D, 5% bottom panel shows a cluster of Pax8+ cells).

**Figure 5.**
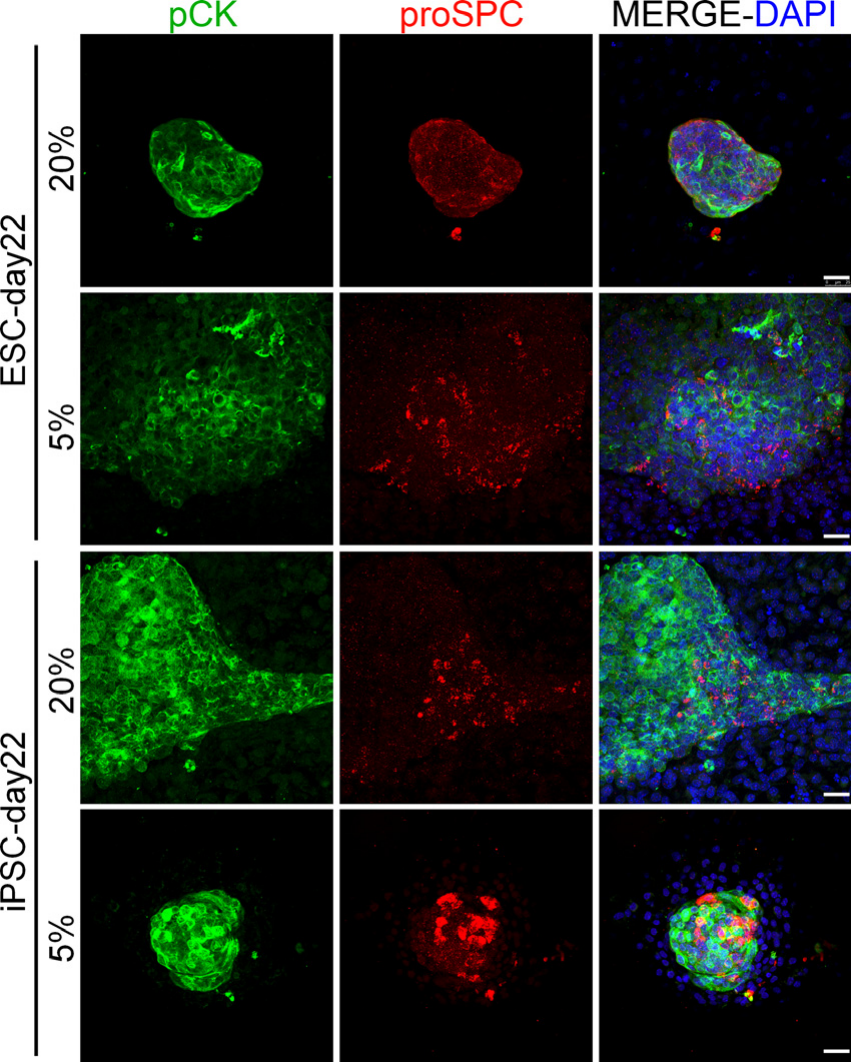
Representative images of double immunofluorescence on day 22 of the protocol against pan‐cytokeratin (pCK) and pro‐surfactant protein C (proSPC) in ESC‐ and iPSC‐differentiated cultures, under 20% and 5% oxygen conditions. Scale bar is 25 *μ*m. ESC, embryonic stem cells; iPSC, induced pluripotent stem cells.

Then, we sought to quantify the Foxa2+Nkx2.1+Pax8‐ cells in our cultures, considered as progenitor cells competent to undergo lung commitment. With this purpose, we analyzed three independent experiments by triple immunofluorescence staining against Nkx2.1, Foxa2, and Pax8 and counted the Foxa2+Nkx2.1+Pax8‐ cells. On day 12, 5% oxygen tension improved the generation of Foxa2+Nkx2.1+Pax8‐ cells in both ESC (1.5‐fold increase compared to 20% oxygen) and iPSC (30‐fold increase compared to 20% oxygen) differentiated cultures (Fig. 4E and F).

**Figure 6.**
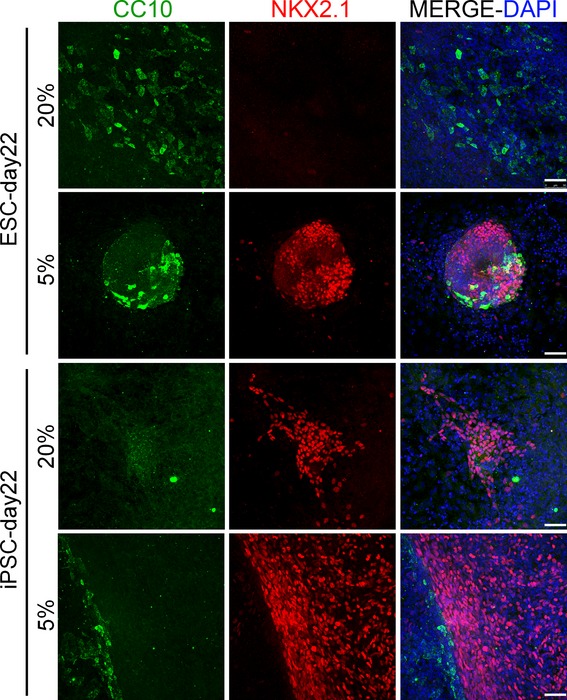
Representative images of double immunofluorescence on day 22 of the protocol against clara cell secretory protein (CC10) and NKX2.1 in ESC‐ and iPSC‐differentiated cultures, under 20% and 5% oxygen conditions. Scale bar is 50 *μ*m. ESC, embryonic stem cells; iPSC, induced pluripotent stem cells.

Moreover, in order to verify the differentiation potential of our day 12 differentiated cultures into more mature lung epithelial cells, we left the cultures under lung maturation conditions for 10 days more. On day 22, type‐II alveolar epithelial‐like cells were identified by the coexpression of pro‐surfactant protein C and cytokeratin (markers for type‐II alveolar epithelial cells) in ES and iPS differentiated cultures at both oxygen conditions (Fig. 5). In addition, cells expressing clara secretory protein CC10, a marker for clara cells of the proximal airway (Morrisey and Hogan 2010; Garcia et al. 2012; Mou et al. 2012), were detected in ES and iPS differentiated cultures on day 22 at both oxygen conditions (Fig. 6). Interestingly, cell clusters expressing the lung progenitor marker Nkx2.1 were also found on day 22 in ES and iPS differentiated cultures at both oxygen conditions (although qualitatively they were more present at 5% oxygen conditions), indicating that a pool of lung progenitor cells were still maintained (Fig. 6). Overall, these data suggested that Foxa2+Nkx2.1+Pax8‐ lung progenitors within our cultures were able to give rise to mature lung phenotypes.

## Discussion

The control of the pluripotency of ES and iPS cells and their guided differentiation toward specific cell types are major hurdles for their successful use in future clinical applications. There is increasing evidences that oxygen regulates stem cell potency and differentiation. Low oxygen tension favors the establishment of new mouse and human ESC lines from blastocysts (Ludwig et al. 2006; Wang et al. 2006). In the same manner, low oxygen has been proven to facilitate the reprogramming of somatic cells to iPSC (Yoshida et al. 2009; Shimada et al. 2012). Moreover, differentiation of pluripotent stem cells under low oxygen tension has been shown to enhance the generation of a variety of cell phenotypes, including neuronal (Fernandes et al. 2010; Garita‐Hernández et al. 2013; Stacpoole et al. 2013; Binh et al. 2014), cardiac (Ng et al. 2010; Van Oorschot et al. 2011; Horton and Auguste 2012), endothelial (Han et al. 2010; Prado‐Lopez et al. 2010; Shin et al. 2011), hematopoietic (Lesinski et al. 2012), and chondrogenic (Koay and Athanasiou 2008; Adesida et al. 2012) cells among others. Therefore, oxygen is becoming a key signaling molecule which has to be taken into account in the designing of efficient strategies to direct stem cell differentiation. Here, we provide new evidence that the oxygen level also modulates the generation epithelial lung cell lineages from pluripotent stem cells. Specifically, we found that Nkx2.1 and Foxa2 transcription factors were greatly upregulated at 5% oxygen. Moreover, quantification of Foxa2+Nkx2.1+Pax8‐ lung progenitor cells in both ESC and iPSC‐derived cultures demonstrated a positive effect of the 5% oxygen condition. Further differentiation of these cell cultures confirmed their competence to generate more mature lung epithelial cell phenotypes expressing proSPC and CC10 proteins.

The cellular responses to oxygen changes are mediated through the hypoxia‐inducible factor (HIF) family of transcriptional regulators. The HIF transcriptional complex is a heterodimer composed of one of three α‐subunits (HIF‐1α, HIF‐2α, or HIF‐3α) and a β‐subunit (Groenman et al. 2007). Under hypoxic conditions, the α‐subunit is stable and accumulates in the nucleus where, upon binding to the β‐subunit, it recognizes HIF‐response elements within the promoter regions of many hypoxia‐responsive target genes involved in the control of angiogenesis, glucose metabolism, and cellular proliferation. Conversely, under normoxia, the α‐subunit is rapidly degraded (Groenman et al. 2007).

HIF‐1α and HIF‐2α play critical roles in the regulation of lung function and hypoxia‐induced pulmonary vascular remodeling. HIF‐1α knockout mice suffer from severe deficiencies associated with defects in VEGFA expression and vasculogenesis, and die in utero (Compernolle et al. 2003; Saini et al. 2008). The lungs of these mice exhibited impaired alveolar epithelial differentiation and an almost complete loss of surfactant protein expression (Saini et al. 2008). HIF‐2α knockout mice suffer from postnatal respiratory distress due to insufficient surfactant production (Compernolle et al. 2002).

Moreover, HIF‐1α has been investigated as an important gene mediating pluripotent stem cells response to hypoxia, and its implication on self‐renewal and differentiation is reviewed elsewhere (Lee et al. 2012). One of the main downstream target genes of HIF‐1α is VEGF, which is known to coordinate the proper development of lung epithelium and vasculature (Van Tuyl et al. 2005; Zhao et al. 2005). In a lung renal capsule allograft model, that follows closely lung development, Zhao et al. (2005) showed that inhibition of VEGF activity resulted in reduced epithelial proliferation, but permitted lung epithelial differentiation. Also, Lee et al. (2012) have demonstrated in a recent report that hypoxic priming of mouse EBs highly increased the HIF‐1‐mediated VEGF, and was sufficient for efficiently differentiating mouse ESC to endothelial cells without the need of adding exogenous growth factors. Thus, VEGF is involved in lung epithelium proliferation in vivo (Zhao et al. 2005) and in endothelial cell differentiation from ESC in vitro (Lee et al. 2012). Our present data indicate a positive effect of low oxygen exposure on pulmonary differentiation of mouse pluripotent stem cells, showing an increase on lung progenitor numbers derived under 5% oxygen tension. Given the important role of VEGF in lung morphogenesis (Van Tuyl et al. 2005; Zhao et al. 2005), one possible explanation for this low‐oxygen‐mediated increase in lung progenitors could be the induction of VEGF expression, triggered by HIF‐1α, in our cell differentiating cultures. Enhanced VEGF signaling could create a microenvironment in which endothelial progenitor cells could be also generated together with the pulmonary epithelial progenitors. These endothelial progenitors might act in a paracrine route by producing secreted factors, cell surface or extracellular matrix molecules that might provide cues for an enhanced growth of the lung epithelial progenitors. However, additional studies will be needed to elucidate the exact mechanism by which oxygen produces such impact on the generation of early lung progenitors. Furthermore, important parameters such as the hierarchy of cell stemness when exposed to hypoxia and the duration of hypoxic exposure will be of interest on future studies, in order to gain new insights into the role of oxygen‐mediated signaling pathways on the pulmonary differentiation of pluripotent stem cells.

The finding in this study has a potential application for lung repair/regeneration. Specifically, finding an optimal cell source to seed into acellular lungs is an open question subjected to current research in the field of lung bioengineering (Cortiella et al. 2010; Daly et al. 2012; Jensen et al. 2012; Ghaedi et al. 2013). In view of the data reported here, lung progenitors derived from low oxygen ESC and iPSC differentiation protocols might be a suitable source of cells for repopulating acellular lung scaffolds. However, other studies will be required to thoroughly investigate whether low oxygen produces a permanent effect on the generated lung progenitors, the appropriate time of their harvesting, as well as their functionality in vitro and in vivo.

Although future investigations are necessary to study in detail the molecular mechanism of low‐oxygen‐driven pulmonary differentiation and the functionality of the cells produced, to our knowledge, this is the first study addressing the influence of oxygen tension on the differentiation of mouse ESC and iPSC into respiratory cell types. We showed that differentiation into pulmonary cell fates was improved when the process was carried out in an environment with an oxygen partial pressure of 5% (which is close to tissue normoxia) as compared with the conventional culture conditions of room air (20% oxygen), which in fact corresponds to cell hyperoxia.

## Acknowledgment

EG has been the recipient of an IDIBAPS Postdoctoral Fellowship‐ BIOTRACK, supported by the European Community's Seventh Framework Programme (EC FP7/2007‐2013) under the grant agreement number 229673.

## Conflict of Interest

None declared.
